# Hierarchical highly ordered SnO_2_ nanobowl branched ZnO nanowires for ultrasensitive and selective hydrogen sulfide gas sensing

**DOI:** 10.1038/s41378-020-0142-6

**Published:** 2020-05-04

**Authors:** Li-Yuan Zhu, Kai-Ping Yuan, Jia-He Yang, Cheng-Zhou Hang, Hong-Ping Ma, Xin-Ming Ji, Anjana Devi, Hong-Liang Lu, David Wei Zhang

**Affiliations:** 1 State Key Laboratory of ASIC and System, Shanghai Institute of Intelligent Electronics & Systems, School of Microelectronics, Fudan University, 200433 Shanghai, China; 2 Inorganic Materials Chemistry, Ruhr-University Bochum, 44780 Bochum, Germany

**Keywords:** Sensors, Nanowires

## Abstract

Highly sensitive and selective hydrogen sulfide (H_2_S) sensors based on hierarchical highly ordered SnO_2_ nanobowl branched ZnO nanowires (NWs) were synthesized via a sequential process combining hard template processing, atomic-layer deposition, and hydrothermal processing. The hierarchical sensing materials were prepared in situ on microelectromechanical systems, which are expected to achieve high-performance gas sensors with superior sensitivity, long-term stability and repeatability, as well as low power consumption. Specifically, the hierarchical nanobowl SnO_2_@ZnO NW sensor displayed a high sensitivity of 6.24, a fast response and recovery speed (i.e., 14 s and 39 s, respectively), and an excellent selectivity when detecting 1 ppm H_2_S at 250 °C, whose rate of resistance change (i.e., 5.24) is 2.6 times higher than that of the pristine SnO_2_ nanobowl sensor. The improved sensing performance could be attributed to the increased specific surface area, the formation of heterojunctions and homojunctions, as well as the additional reaction between ZnO and H_2_S, which were confirmed by electrochemical characterization and band alignment analysis. Moreover, the well-structured hierarchical sensors maintained stable performance after a month, suggesting excellent stability and repeatability. In summary, such well-designed hierarchical highly ordered nanobowl SnO_2_@ZnO NW gas sensors demonstrate favorable potential for enhanced sensitive and selective H_2_S detection with long-term stability and repeatability.

## MEMS: sensing hydrogen sulfide with nanobowl branched nanowires

High performance hydrogen sulfide detection is demonstrated for SnO_2_ nanobowls with branched ZnO nanowires, in-situ fabricated on a MEMS device. Hydrogen sulfide is a particularly hazardous substance to human health, and its detection via MEMS has a number of advantages in practical applications. However, coating the sensing elements directly on to a device is challenging, and often leads to insufficient reliability. Now, a team led by Hong-Liang Lu from Fudan University demonstrate atomic layer deposition of ZnO film onto SnO_2_ nanobowls, followed by ZnO nanowire growth to create a highly branched structure as the sensing element. The process is completed on the MEMS device itself. High sensitivity and fast response is in part attributed to the large surface area of the branched nanowires, and performance is stable for a month.

## Introduction

Hydrogen sulfide (H_2_S), one of the most dangerous hazardous gases, has aroused widespread concern for its severe toxicity to the human body as well as being normally generated from industries^1–3^. Trace levels of H_2_S are sufficient to damage the human respiratory system as well as cause unconsciousness neurological sequelae and cardiovascular-related death^1^. In view of this, it is of great significance to effectively detect and monitor H_2_S in the surrounding living environment. To date, diverse chemical sensors based on different mechanisms have been extensively investigated and developed to detect trace levels of H_2_S, including chemiresistive^4,5^, electrochemical^6^, and optical^7^ sensors. Among these, semiconducting metal oxide (SMO)-based chemiresistive sensors have attracted tremendous research interest due to their irreplaceable advantages of low cost, convenient fabrication, and great integrated circuit compatibility^8^.

Among various investigated SMOs, tin-oxide (SnO_2_) nanomaterials with different morphologies, such as nanoparticles^9^, nanowires (NWs)^10^, nanofibers^11^, and nanobamboos^12^, have been intensively explored and regarded as the most promising candidates for high-performance gas sensors, with numerous merits, such as high carrier mobility, great chemical and thermal stability, and low cost^13,14^. However, these nanostructures are usually brush printed or drop coated onto ceramic tubes or microelectromechanical system (MEMS) devices, which intrinsically limits the reliability and repeatability of the fabricated sensors^15^. Therefore, a new strategy of seamlessly integrating nanomaterials and microhotplatforms for the sensor fabrication process is of vital importance for obtaining high-performance gas sensors with excellent stability and repeatability, as well as low power consumption. Very recently, a hard template method was developed for in situ monolayer macroporous material synthesis, which fits the requirements mentioned above^16^. The in situ preparation process could not only benefit wafer-level fabrication but also effectively reduce the contact resistance and improve the device performance. For instance, Gu et al. prepared an ordered macroporous structure of SnO_2_ by employing a monolayer polymethyl methacrylate (PMMA) sphere template, which exhibited excellent repeatability with ethanol and had a promising application in ethanol detection^17^. Additionally, such a highly ordered monolayer macroporous structure demonstrated enhanced gas response and response speed due to its large specific surface area and well-interconnected pore structure^18^.

However, pristine SnO_2_ gas sensors usually suffer from problems such as poor selectivity and long response-recovery time^17^. Accordingly, diverse effective approaches have been developed to improve the performance of gas sensors, such as noble metal doping^19,20^, hierarchical structure construction^21^, and composite heterostructure design^22,23^. In particular, the construction of hierarchical structures is beneficial for increasing the specific surface area and forming more nanojunctions at the interface between the initial and secondary nanostructures and has been widely regarded as one of the most promising strategies and has stimulated great research interest. For example, Alenezi et al. synthesized hierarchical zinc oxide (ZnO) NWs and nanodisks assembled from initial ZnO nanostructures following a hydrothermal process and demonstrated an enhancement in the acetone sensing performance in comparison to the initial ZnO monomorphological nanostructures^24^. Furthermore, heterogeneous-hierarchical nanocomposites exhibited superior gas sensing performance than homohierarchical nanostructures considering the synergistic effect of various properties from different materials. Zhang et al. successfully synthesized novel brush-like SnO_2_@ZnO hierarchical nanostructures with SnO_2_ NW backbones and ZnO nanorod branches via a simple two-step hydrothermal method and demonstrated that the nitrogen dioxide (NO_2_) sensing performance of SnO_2_@ZnO hierarchical structures was substantially enhanced at a relatively low operating temperature (150 °C) in comparison to that of pristine ZnO and SnO_2_^25^.

Herein, a novel synthetic route for the large-scale fabrication of hierarchical highly ordered SnO_2_ nanobowl branched ZnO NWs with excellent H_2_S sensing performance is proposed combining a hard template method for the preparation of highly ordered SnO_2_ nanobowls, atomic-layer-deposition (ALD) processing of nanoscale ZnO seed layers on the surface of SnO_2_ nanobowls, and modified hydrothermal processing for the growth of branched ZnO NWs. In particular, the ALD technique can precisely control the film thickness and provide uniform conformal coverage of ZnO seed layers on SnO_2_ nanobowls^26^. The morphology of the hierarchical structures was tuned by the thickness of the ALD-controlled seed layers and the hydrothermal growth time. The hierarchical branches grown on highly ordered nanobowls can provide an enlarged specific surface area for gas adsorption, as well as efficient channels for electron transport, compared to that of the ZnO film shell^27^. More importantly, hierarchical sensing materials were synthesized in situ on MEMS devices, which are expected to be high-performance gas sensors with superior sensitivity, long-term stability and repeatability, as well as low power consumption. As a result, the substantially enhanced sensing performance of hierarchical nanobowl SnO_2_@ZnO NW gas sensors to H_2_S compared to the pristine SnO_2_ nanobowl sensor and the heterostructured nanobowl SnO_2_@ZnO film sensor was demonstrated. Specifically, the hierarchical nanobowl SnO_2_@ZnO NW sensor displayed a high sensitivity (R_a_/R_g_) of 6.24, a fast response and recovery speed (i.e., 14 s and 39 s, respectively), and an excellent selectivity when detecting 1 ppm H_2_S at 250 °C, and its rate of resistance change (i.e., 5.24) is 2.6 times higher than that of the pristine SnO_2_ nanobowl sensor. The mechanisms of substantially improved sensing performance were proven by both electrochemical characterization and band alignment analysis, suggesting the synergistic effect of the hierarchical heterostructures, including increased specific surface area, the formation of heterojunctions and homojunctions, as well as the additional reaction between ZnO and H_2_S. Moreover, the well-structured hierarchical sensors maintained stable performance after a month, suggesting great stability and repeatability. The combined diversity of hierarchical heterogeneous nanocomposites with low-power MEMS holds favorable potential for highly sensitive and selective H_2_S gas sensors with long-term stability and repeatability.

## Results and discussion

**Fig. 1:**
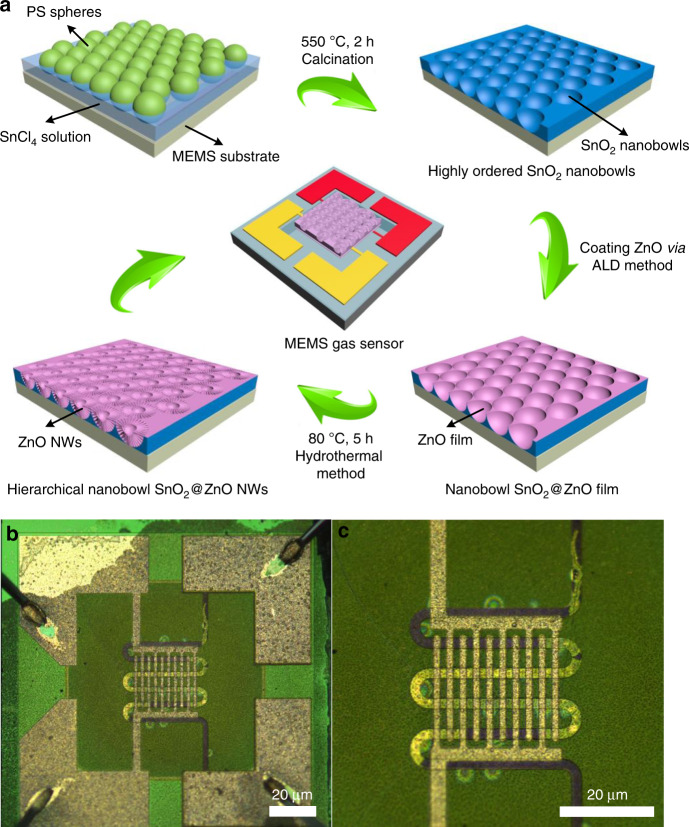
Synthesis of the hierarchical highly ordered nanobowl SnO2@ZnO NWs in situ on MEMS. **a** The synthetic protocol for the hierarchical highly ordered nanobowl SnO_2_@ZnO NWs in situ on MEMS, combining a modified facile hard template method, an ALD process and a hydrothermal method; **b** the optical microscopy image of the MEMS heating appliance with gas sensing materials; **c** an enlarged image of heating electrodes and interdigital sensing electrodes

**Fig. 2:**
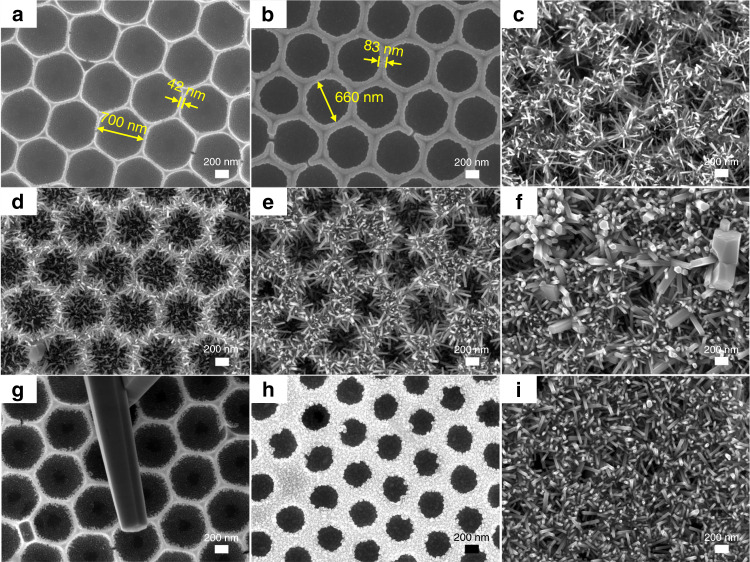
SEM characterization of all the samples. **a** Pristine highly ordered SnO_2_ nanobowls (i.e., S@Z0); **b** highly ordered SnO_2_ nanobowls coated with 20nm ZnO film (i.e., S@Z20); **c** highly ordered SnO_2_ nanobowls branched ZnO NWs (i.e., S@Z20-Z5); **d** S@Z20-Z1; **e** S@Z20-Z3; **f** S@Z20-Z8; **g** S@Z0-Z5; **h** S@Z10-Z5; **i** S@Z30-Z5

Figure 1a illustrates the synthetic protocol of the hierarchical highly ordered nanobowl SnO_2_@ZnO NWs in situ on MEMS combined with the ALD process. First, highly ordered monolayer polystyrene (PS) spheres soaked with SnCl_4_ precursor solution were obtained on the MEMS substrate *via* a simple picking-up process. After thorough drying at room temperature and calcination in a muffle furnace at 550 °C for 2 h, the organic PS spheres were removed, and a uniform and shiny highly ordered SnO_2_ nanobowl film could be seen on the surface of the MEMS substrate. Figure 1b, c and Fig. S1 show the optical microscopy and SEM images of the MEMS substrate with the obtained materials, displaying the whole structure of the MEMS gas sensor as well as demonstrating the intactness of the device after 550 °C calcination. Figure 2a shows the top-view SEM image of a randomly selected area of the SnO_2_ nanobowl film, confirming that the film is composed of highly ordered SnO_2_ nanobowls with a diameter of ~700 nm. It is noteworthy that the diameter of the nanobowls is slightly shorter than the diameter of the PS template, which reveals that the SnCl_4_ precursor solution level was lower than the half height of the PS sphere. Moreover, since the PS spheres were closely packed on the substrate, an approximate triangular space was naturally formed among three adjacent spheres, exhibiting hexagonal close packing. Then, these highly ordered SnO_2_ nanobowls were conformably coated by ZnO seed layers with different thicknesses through thermal ALD at 200 °C. The displayed SEM image in Fig. 2b indicates the as-prepared highly ordered nanobowl SnO_2_@ZnO film morphology with a uniform and conformal ZnO seed layer. The average thickness of the ZnO seed layer turned out to be 20 nm with 100 ALD growth cycles, which is consistent with the result of the ZnO film grown on the flat Si wafer measured by the SE system. In addition, the thicknesses of the ZnO films with 50 and 150 ALD growth cycles were measured to be 10 and 30 nm, respectively. Evidently, the thickness of the ZnO film shows a continuous increase with a slope of ~0.2 nm/cycle when increasing the total ALD growth cycles. The corresponding samples are denoted as S@Z*X* in this work, where *X* = 0, 10, 20, and 30, representing the thicknesses of the ZnO films. Finally, owing to the uniform and conformal coverage of the ZnO seed layer, the ZnO NWs were successfully grown on the surface of the S@Z20 structure via a modified hydrothermal reaction. The ZnO NWs grew densely on the surface of S@Z20, with the highly ordered nanobowl morphology of S@Z20 remaining, appearing similar to a nest-like array (Fig. 2c). The optimal hydrothermal reaction time was further investigated to obtain hierarchical highly ordered nanobowl SnO_2_@ZnO NWs with an excellent morphology, which have as large a specific surface area as possible on the basis of maintaining a complete hierarchical structure. Similarly, the corresponding hierarchical samples are denoted as S@Z20-Z*Y*, where *Y* = 1, 3, 5, and 8, representing the hydrothermal reaction time.

The morphologies of S@Z20-Z*Y* (*Y* = 1, 3, 5, and 8) displayed regular changes with an increase in hydrothermal reaction time. At 1 h (Fig. 2d), the branched ZnO NWs were relatively short, and overall, the hierarchical S@Z20-Z1 retained a macroporous morphology. As shown in Fig. 2d, e and Fig. 2c, when the hydrothermal reaction time is increased from 1 to 5 h (i.e., S@Z20-Z1, S@Z20-Z3, and S@Z20-Z5), the length of branched ZnO NWs is significantly increased, and the whole hierarchical structure seems to be denser. As the hydrothermal reaction time reached 5 h, sample S@Z20-Z5 achieved a maximum length of branched ZnO NWs and maintained a complete hierarchical highly ordered nanobowl branched nanowire structure (Fig. 2c). However, when the hydrothermal reaction time further increased to 8 h, as shown in Fig. 2f, the SnO_2_ nanobowls were fully filled with ZnO NWs, which damaged the designed morphology and greatly limited the effective function of SnO_2_ nanobowls. In addition, the crystalline grain of ZnO NWs showed a significant increase (Fig. 2f), reducing the specific surface area. As a result, the optimal hydrothermal reaction time for branched ZnO NWs growing on highly ordered SnO_2_ nanobowls is 5 h.

Moreover, the effect of the seed layer thickness on the hierarchical nanowire-branched nanobowl structure was further investigated with a determined hydrothermal reaction time of 5 h. Figure 2g-i displays the morphologies of the samples S@Z0-Z5, S@Z10-Z5, and S@Z30-Z5. When the highly ordered SnO_2_ nanobowls without a ZnO seed layer were treated with a 5 h hydrothermal process, branched ZnO NWs could not grow on the surface of the nanobowls, and instead, there would only be nanowires with large grain sizes physically adsorbed on the surface of S@Z0-Z5 across several nanobowls (Fig. 2g). Hence, a uniform and conformal seed layer deposited by ALD is of vital importance to the synthesis of hierarchical structures with branched nanowires. In addition, the thickness of the seed layer would specifically affect the entire hierarchical morphology. It was demonstrated that the highly ordered SnO_2_ nanobowls coated with a moderate-thickness ZnO seed layer (i.e., S@Z20-Z5) exhibited the optimal hierarchical structure (Fig. 2c). However, if the seed layer was much thinner (i.e., S@Z10-Z5), there would only be ZnO nanograins on the surface of the seed layer, which were not able to grow into nanowires (Fig. 2h). On the other hand, if the seed layer was too thick (i.e., S@Z30-Z5), the branched ZnO NWs grown *via* the 5 h hydrothermal process would completely fill the nanobowls, thus destroying the designed hierarchical structure (Fig. 2i).

**Fig. 3:**
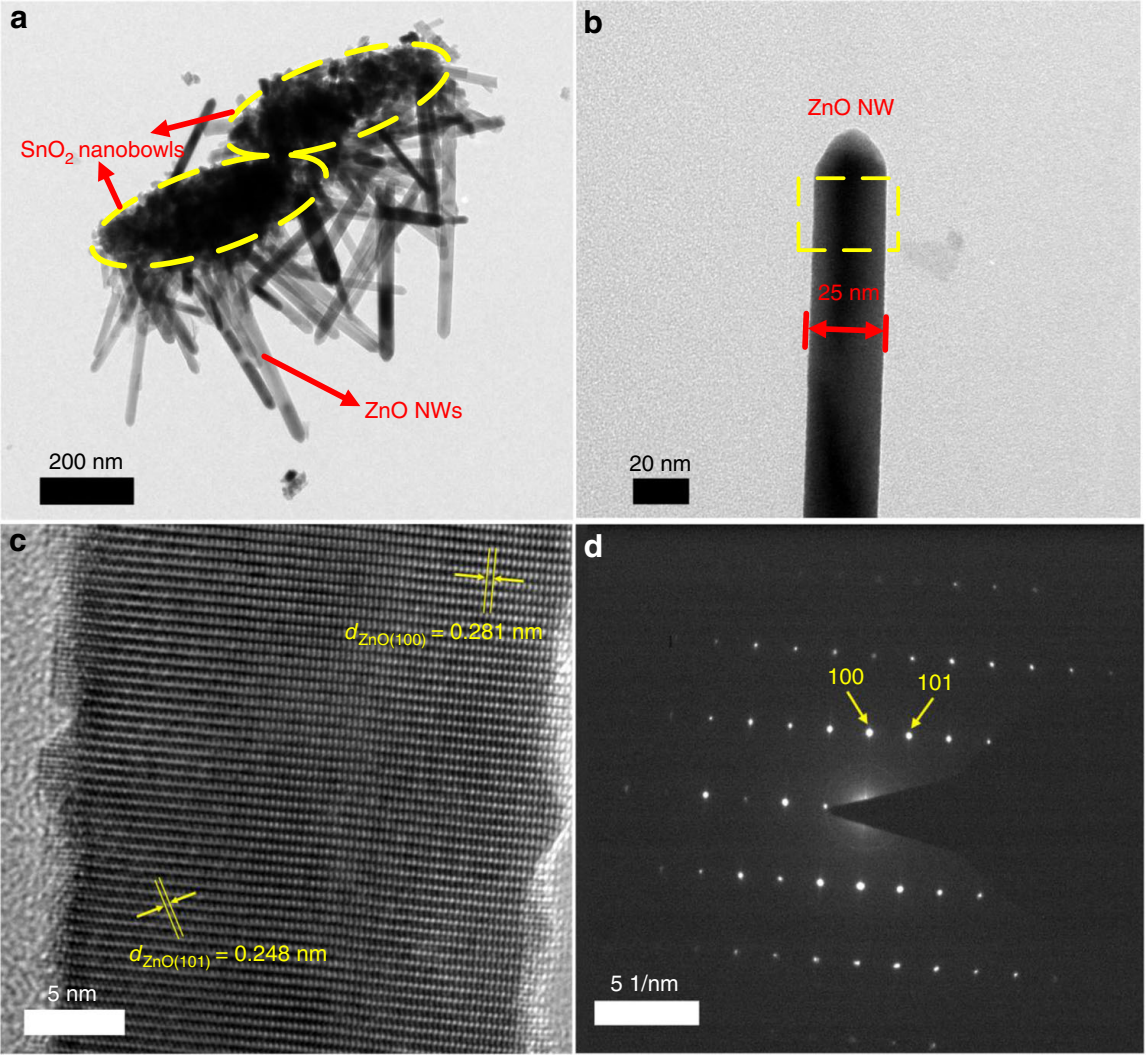
TEM characterization of the highly ordered SnO2 nanobowl branched ZnO NW sample S@Z20-Z5. **a** Low-magnification image of two nanowire-branched nanobowls; **b** the highly magnified image of a randomly selected single ZnO NW on S@Z20-Z5; **c** the HRTEM image and **d** the SAED pattern obtained on a single ZnO NW

The well-defined hierarchical nanowire-branched nanobowl structure could be further revealed by TEM characterization on a typical sample S@Z20-Z5 (Fig. 3). Figure 3a shows the bright-field TEM image of two single S@Z20-Z5 nanowire-branched nanobowls, clearly displaying the grafting of ZnO NWs on the surface of SnO_2_ nanobowls, which is consistent with the SEM results. A highly magnified TEM image (Fig. 3b) indicates the complete morphology and smooth surface of a randomly selected single ZnO NW with a diameter of ~25 nm. The high-resolution TEM (HRTEM) image (Fig. 3c) and the selected-area electron diffraction (SAED) pattern (Fig. 3d) of the randomly selected single ZnO NW on sample S@Z20-Z5 reveal that the branched ZnO nanowires have a single-crystalline structure. The lattice fringes of 0.248 nm and 0.281 nm in the single ZnO NW could be clearly identified, which correspond to the *d*-spacing values of the (101) and (100) planes of the hexagonal wurtzite ZnO phase (PDF#36–1451), respectively.

**Fig. 4:**
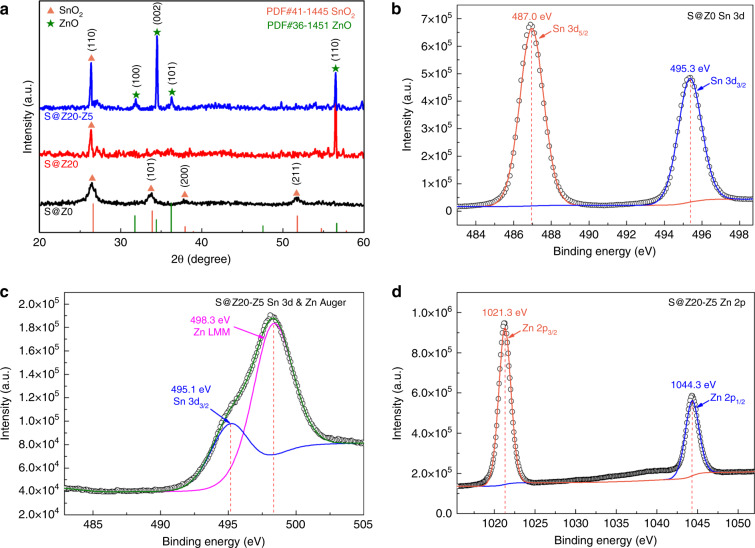
Chemical components of S@Z0, S@Z20, and S@Z20-Z5. **a** XRD patterns of S@Z0, S@Z20, and S@Z20-Z5; **b** high-resolution core level Sn 3d spectrum of the highly ordered SnO_2_ nanobowl sample S@Z0; **c** high-resolution core level Sn 3d and Zn Auger spectrum and **d** high-resolution core level Zn 2p spectrum of the highly ordered SnO_2_ nanobowl branched ZnO NWs sample S@Z20-Z5

As shown in Fig. 4a, the crystal structures of hierarchical highly ordered SnO_2_ nanobowl branched ZnO NWs were investigated by XRD. Regarding pristine SnO_2_ nanobowls, after annealing at 550 °C in air, the measured diffraction peaks could be well indexed into a cassiterite SnO_2_ phase (PDF#41–1445). Specifically, the S@Z0 sample showed four characteristic diffraction peaks at 26.6°, 33.9°, 37.9°, and 51.8°, which correspond to the (110), (101), (200), and (211) planes of the cassiterite structure of SnO_2_, respectively. Compared to the pristine SnO_2_ nanobowls, the S@Z20 core-shell nanobowls obtained after the ALD-ZnO reaction displayed a new strong characteristic diffraction peak at 56.6°, which belongs to the zincite ZnO phase (PDF#36–1451). In addition, three weak diffraction peaks belonging to the SnO_2_ phase were not detected in the S@Z20 sample, which may be affected by the coating of the 20-nm ZnO film. Subsequently, ZnO NWs were further grafted onto the surface of S@Z20 via a hydrothermal process (i.e., S@Z20-Z5), and three additional peaks at 31.7°, 34.4°, and 36.2° were detected, further confirming the hexagonal wurtzite structure of ZnO. Moreover, the prominent ZnO (002) peak indicated that the branched ZnO NWs had a preferential growth orientation along the *c*-axis. It should be noted that no other impurity peak was found in the XRD patterns, which confirmed the phase purity of the prepared samples.

Figure 4b-d and Figure S3 display the XPS spectra of pristine highly ordered SnO_2_ nanobowls (i.e., S@Z0) and sample S@Z20-Z5. The full spectrum of S@Z0 in Fig. S3a simply shows the peak positions of Sn 3d and O 1 s, revealing the exact SnO_2_ composition. By contrast, as shown in Fig. S3c, the additional peaks of Zn 2p and Zn LMM in the full spectrum of S@Z20-Z5 confirm the presence of ZnO in such a heterostructured sample. Specifically, the detailed XPS spectrum of Sn 3d in S@Z0 (Fig. 4b) displays two distinct peaks at binding energies of 487.0 and 495.3 eV, corresponding to the Sn 3d_5/2_ and Sn 3d_3/2_ core levels, respectively. For the detailed XPS spectrum of Sn 3d in S@Z20-Z5 (Fig. 4c), the asymmetric peak could be coherently fitted by two nearly Gaussian components, centered at 495.1 and 498.3 eV. The peak located at 487.0 eV could be assigned to the characteristic Sn 3d_5/2_ peak, which is identical to the typical value of SnO_2_. However, the other peak at a higher binding energy of 498.3 eV could be attributed to the Auger electron peak of Zn LMM, demonstrating the existence of Zn in the sample^28^. Moreover, the Zn 2p XPS spectrum (Fig. 4d) of sample S@Z20-Z5 exhibits the characteristic Zn 2p_3/2_ and Zn 2p_1/2_ peaks centered at 1021.3 and 1044.3 eV, respectively, which confirms the presence of ZnO composition. The detailed O 1 s spectra of S@Z0 and S@Z20-Z5 can be found in Fig. S3b and d. Both asymmetric O 1 s peaks could be deconvoluted into two parts, assigned to oxygen in the SnO_2_ or ZnO crystal lattice and the surficial adsorbed oxygen, respectively^29^.

**Fig. 5:**
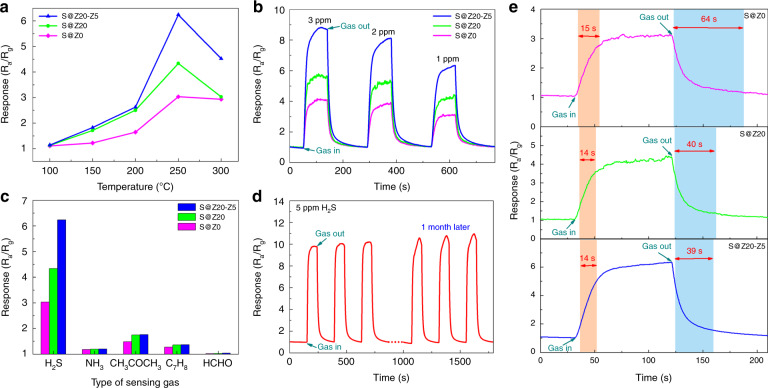
Gas sensing properties of the three samples (i.e., S@Z0, S@Z20, and S@Z20-Z5). **a** Response of all three samples at different operating temperatures (i.e., 150, 200, 250, and 300°C); **b** dynamic response curves of all three samples with different hierarchical structures facing the reducing gas of H_2_S with various concentrations ranging from 3 to 1 ppm at 250°C; **c** response of all three samples facing other various reducing gases (1 ppm), namely, NH_3_, CH_3_COCH_3_, C_7_H_8_, and HCHO, compared with 1 ppm H_2_S at 250°C; **d** the characterization of long-term stability for sample S@Z20-Z5 in air for a month; **e** enlarged responses of samples S@Z0, S@Z20, and S@Z20-Z5 facing 1 ppm H_2_S at 250°C with fast response and recovery

The prepared samples were all synthesized in situ on MEMS structures to form the proposed gas sensors, as shown in Fig. 1. The JF02F gas sensing measurement system was used to evaluate the gas sensing properties of all the sensors. Typically, three representative MEMS-based sensors (i.e., sample S@Z0, S@Z20, and S@Z20-Z5) were chosen as examples for further investigation of gas sensing performance. The S@Z0 sample was only a highly ordered SnO_2_ nanobowl monolayer film with neither ZnO seed layers nor a hierarchical branched nanowire structure. The S@Z20 sample had a 20-nm-thick seed layer of ZnO film, and the S@Z20-Z5 sample had further branched ZnO NWs with optimal length and diameter on the basis of a ZnO seed layer. First, the response of a gas sensor is greatly affected by the operating temperature. Hence, the relationship between the gas sensing response and the operating temperature was first investigated in Fig. 5a. For all the sensors, their gas sensing responses continued to grow as the operating temperature increased from 100 to 250 °C. All the sensors yielded maximum responses at 250 °C, indicating that the optimal operating temperature for the designed materials in this work could be chosen as 250 °C. However, all the responses began to decrease as the operating temperature further increased above 250 °C (i.e., 300 °C). H_2_S molecules will have difficulty reacting with adsorbed oxygen at a low operating temperature but will desorb before the reaction can occur at an excessively high operating temperature^18^. In addition, the diffusion rate of H_2_S molecules adsorbed on the material surface will improve with increasing temperature^30^. Therefore, at the optimal operating temperature of 250 °C, the gas sensing responses achieved maxima. The S@Z20-Z5 sensor displayed the highest response at any temperature.

Figure 5b shows the corresponding dynamic gas sensing response curves of all the samples (i.e., sample S@Z0, S@Z20, and S@Z20-Z5) with different hierarchical structures at the optimal operating temperature of 250 °C, obtained for the reducing gas of H_2_S with various concentrations ranging from 3 to 1 ppm. Apparently, the responses of all samples decrease as the H_2_S concentration decreases. More importantly, compared with the pristine highly ordered SnO_2_ nanobowl gas sensor (i.e., S@Z0), the designed hierarchical nanobowl SnO_2_@ZnO NW gas sensor (i.e., S@Z20-Z5) does exhibit superior gas sensing performance, which may be closely related to both the construction of the heterostructure and the effective increase in the specific surface area. Specifically, when the H_2_S concentration was fixed to 1 ppm, the response of S@Z20-Z5 was approximately 6.24, whose rate of resistance change (~5.24) was 2.6- and 1.6-fold those of the S@Z0 (~2.03) and S@Z20 (~3.34) sensors, respectively. Table 1 presents the sensing performance comparison of the prepared S@Z20-Z5 sensor with various other SnO_2_-based gas sensors fabricated on MEMS^31–36^. Comprehensively considering the related factors, including operating temperature, response values and response/recovery time, the S@Z20-Z5 sensor shows improved performance among the MEMS-type SnO_2_-based gas sensors.

**Table 1:** Sensing performance comparison of various SnO_2_-based gas sensors fabricated on MEMS

Materials	Target gas	Response (R_a_/R_g_)	Operating temperature [°C]	Response time [s]	Recovery time [s]
Porous SnO_2_ 3D architectures^31^	HCHO (0.5 ppm)	2.9	240	/	/
SnO_2_-ZnO composite nanofibers^32^	NO_2_ (50 ppm)	3.3	25	264	294
Hierarchical SnO_2_-ZnO NWs^33^	C_2_H_5_OH (25 ppm)	3.0	400	/	/
SnO_2_/ZnO core-shell nanosheets^34^	C_2_H_5_OH (100 ppm)	13.3	350		
Hierarchical SnO_2_-ZnO nanofibers^35^	CH_3_OH (2 ppm)	3.4	250	18	25
Porous SnO_2_-ZnO hybrid nanotubes^36^	H_2_S (1 ppm)	4.29	220	93	205
Hierarchical nanobowl SnO_2_@ZnO NWs (this work)	H_2_S (1 ppm)	6.24	250	14	39

To investigate the time dependence of the response, Fig. 5e shows the transient responses of all three samples exposed to 1 ppm H_2_S at 250 °C. The response time and recovery time were measured as the time taken for the sensor output to change from 10 to 90% of the highest response variation. The recovery time of S@Z20-Z5 is 39 s, which obviously exhibits a better recovery property than the pristine S@Z0 sensor (i.e., 64 s). However, the recovery time of S@Z20-Z5 (i.e., 14 s) is slightly shorter than that of S@Z0 (i.e., 15 s). This indicates that compared with the pristine S@Z0 sensor, the S@Z20-Z5 sensor exhibits a faster response when exposed to H_2_S gas. At the same time, the S@Z20-Z5 sensor possesses a better recovery property when turning off the gas and flushing with air. Actually, the existence of the SnO_2_@ZnO heterojunction will improve the hole-electron separation rate at the interface, therefore improving the response and recovery speed to some extent^37^.

In practice, good selectivity is a critical factor for a gas sensor, especially for distinguishing the given target gas from a complex atmosphere. Herein, the selectivity of the S@Z20-Z5 sensor was studied towards H_2_S and various other reducing gases (1 ppm), such as ammonia (NH_3_), acetone (CH_3_COCH_3_), methylbenzene (C_7_H_8_), and formaldehyde (HCHO), at an operating temperature of 250 °C, which is shown in Fig. 5c and Fig. S4. It can be observed apparently that the responses of all three samples to H_2_S are significantly higher than those of other reducing gases, especially for the S@Z20-Z5 sensor, confirming the excellent selectivity. Generally, the selectivity is related to various complicated factors. First, H_2_S has a relatively small band dissociation energy of 381 kJ/mol compared with other gases, which promotes decomposition and surface reactions during chemical adsorption at lower temperature^38^. As for the accurate calculation of the corresponding surface reaction kinetics, first-principles calculations based on density functional theory could be applied, which needs to be further investigated^39^. Second, the XPS characterization of the SnO_2_@ZnO heterostructures before and after the H_2_S-sensing test conducted by Fu et al. has demonstrated that ZnO will react with H_2_S and transfer to ZnS, leading to a larger response since the conductivity of ZnS is higher than that of ZnO^40^. Furthermore, the reaction between ZnO and adsorbed H_2_S is an exothermic and spontaneous process, while the reactions between ZnO and other test gases are endothermic and nonspontaneous^41^. Therefore, the introduction of branched ZnO NWs further enhances the selectivity to H_2_S. Finally, H_2_S is considered to possess a larger adsorption capacity on the same surface adsorption area, which is attributed to the relatively small molecular size of H_2_S among these gas molecules. In addition, the S@Z20-Z5 sensor has a better selectivity than the one based on pristine SnO_2_ nanobowls. This phenomenon further confirmed the important role of the proposed branched ZnO NWs in improving the selectivity.

On the other hand, stability is also a key parameter from the viewpoint of practical gas sensing applications. The assessment of long-term stability was carried out on S@Z20-Z5 in ambient air for a month, as shown in Fig. 5d. Obviously, after a month, the S@Z20-Z5 sensor exhibited less than 5% variation in response when exposed to 5 ppm H_2_S, illustrating good long-term stability. However, it is undeniable that the response of the S@Z20-Z5 sensor exhibited a slight increase after a series of H_2_S gas sensing measurements for a month. The slightly higher response after a month may be caused by a few ZnS residual on the surface of the sensing material, which was transformed from ZnO in the presence of H_2_S and had a higher conductivity than ZnO. Fortunately, since the residual ZnS could be retransformed into ZnO more thoroughly at a higher temperature than the operating temperature of 250 °C^42^, the response after a long-term test can return to the original value with the help of a high-temperature thermal treatment. Moreover, the top-view SEM image of a randomly selected area of S@Z20-Z5 after the above long-term tests is shown in Fig. S5. The intact hierarchical nanowire-branched nanobowl structure further demonstrated good long-term stability.

In general, the gas sensing mechanism is based on the surface adsorption/desorption model of oxygen species^43^. It was observed in Fig. S6 that the resistances of all three sensors show a sudden decrease when introducing the reducing gas of H_2_S, indicating that the sensors exhibit typical *n*-type conductivity behaviors according to the acknowledged mechanism for the SMO-based resistance-type gas sensors^44^. Specifically, when the *n*-type sensor was exposed to air, the physically adsorbed oxygen on the surface would capture electrons from the conduction band of sensing materials and turn into chemisorbed oxygen such as O_2_^−^, O_2_^2−^, O^−^ or O^2−^ ^45,46^. Therefore, an electron-depleted region formed underneath the material surface, causing the sensing material to be in a high-resistance state. Once the *n*-type sensor was exposed to the reducing gas of H_2_S, the chemisorbed oxygen species on the surface would react with H_2_S and release the electrons back to the material, as typically shown in Eq. (1)^3^:

1 $${\mathrm{H}}_2{\mathrm{S}} + 3{\mathrm{O}}_{\left( {{\mathrm{ads}}} \right)}^{2 - } \to {\mathrm{SO}}_2 + {\mathrm{H}}_2{\mathrm{O}} + 6{\mathrm{e}}^ -$$

**Fig. 6:**
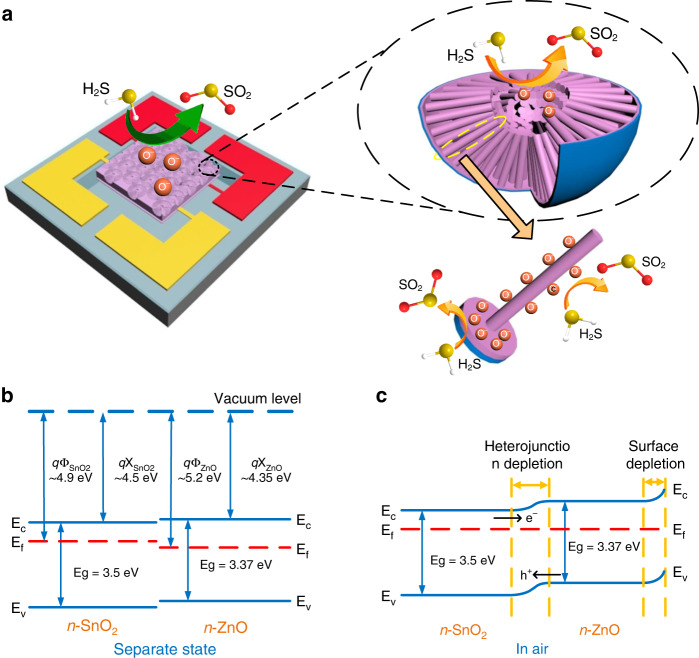
Schematics of the reducing gas sensing mechanism in the hierarchical highly ordered nanobowl SnO2@ZnO NWs. **a** The three-dimensional schematic of the hierarchical highly ordered nanobowl SnO_2_@ZnO NWs exposed in H_2_S gas; **b**, **c** the schematic energy band diagrams for the hierarchical SnO_2_@ZnO NWs: **b** in separate state and **c** in air. The band structure data in figure (**b**) were determined from the literature^48^

Therefore, the resistance of the *n*-type sensor will achieve a rapid decrease when introducing the reducing gas, which is in accordance with the experimental results shown in Fig. S6. The three-dimensional schematic of S@Z20-Z5 exposed to H_2_S gas is shown in Fig. 6a.

The experimental results in Fig. 5b observably show that the sensing properties of the highly ordered SnO_2_ nanobowl branched ZnO NW sensor (i.e., S@Z20-Z5) were much better than that of the pristine SnO_2_ nanobowl sensor (i.e., S@Z0). There are three main factors that satisfactorily account for the enhancement of gas sensing performance. First, due to the introduction of hierarchical branched ZnO NWs, the increase in specific surface area effectively increases the active adsorption sites and enhances the sensing response^24^. To provide direct experimental evidence for the increase in specific surface area, the electrochemically active surface areas of S@Z20 and S@Z20-Z5 were measured through electrical double-layer capacitance (EDLC) measurements in Na_2_SO_4_ solution on the basis that the double-layer capacitance (C_dl_) is proportional to the electrochemically active surface area^47^. The cyclic voltammetry (CVs) were tracked in the range of −0.30 to −0.19 V, where the current response should only refer to the charging of the double layer. The CV curves of the S@Z20 and S@Z20-Z5 electrodes at different scan rates (1, 2.5, 5, 7.5, 10, 15, 20, 25, and 30 mV/s) are shown in Fig. S7a, b, respectively. As a result, the capacitance of the S@Z20-Z5 electrode (0.223 mF/cm^2^, Fig. S7d) is higher than that of the S@Z20 electrode (0.119 mF/cm^2^, Fig. S7c), indicating the higher active surface area of the S@Z20-Z5 electrode, which is consistent with the SEM results. Therefore, on the basis of the CV measurements, the hierarchical heterostructured S@Z20-Z5 sample had a higher specific surface area than S@Z20, which effectively increased the number of gas adsorption sites.

Second, the cause of the resistance modulation could be the formation of the SnO_2_@ZnO heterojunction and ZnO@ZnO homojunction at the corresponding interfaces^33^. The separate band alignment diagrams of SnO_2_ and ZnO are displayed in Fig. 6b. Since the work function of ZnO (5.2 eV) is larger than SnO_2_ (4.9 eV), when the ZnO film as well as ZnO NWs were grown onto highly ordered SnO_2_ nanobowls, the electrons would be transferred from SnO_2_ to ZnO to equalize the Fermi level, leading to the formation of a potential barrier at the *n*-*n* heterojunction interface (Fig. 6c)^48^. As a result, the resistance of the *n*-type S@Z20-Z5 sensor (R_a_) achieved a further increase in air because of the additional existence of a potential barrier formed at the SnO_2_@ZnO heterojunction interface. Correspondingly, when the S@Z20-Z5 sensor was exposed to the reducing gas of H_2_S, the decrease of the heterojunction barrier could help further reduce the resistance (R_g_), leading to the enhancement of the sensing response defined as R_a_/R_g_. Moreover, 20 nm is close to the Debye length of ZnO (~20 nm) at 250 °C, which further benefits the improvement of gas sensing performance^40^. In addition, the ZnO@ZnO homojunction at the contact area of branched ZnO NWs was also attributed to resistance modulation, increasing the resistance in air and further reducing the resistance in reducing gas.

The third factor is the transformation of ZnO to ZnS during the spontaneous reaction with H_2_S^40,41^. When ZnO is exposed to H_2_S, a small amount of ZnO reacts with the adsorbed H_2_S and turns into ZnS on the surface, as shown in Eq. (2):^49^

2 $${\mathrm{ZnO}} + {\mathrm{H}}_2{\mathrm{S}}_{\left( {{\mathrm{ads}}} \right)} \to {\mathrm{ZnS}} + {\mathrm{H}}_2{\mathrm{O}}$$

Since the conductivity of the reaction product ZnS is higher than that of ZnO, the resistance of the whole material would achieve a further decrease after introducing H_2_S and undergoing such a transformation reaction^40^. Correspondingly, when the material was exposed to air again, the small amount of ZnS on the surface reacted with adsorbed oxygen and retransformed into ZnO at the high operating temperature^42^. Therefore, the introduction of ZnO film and branched ZnO NWs further improves the sensitivity of the heterostructured SnO_2_@ZnO sensors. Meanwhile, as mentioned above, the spontaneous reaction between ZnO and H_2_S enhances the selectivity of the heterostructured sensors for H_2_S detection as well.

## Materials and methods

### Chemicals and reagents

Analytical grade tin (IV) chloride hydrate (SnCl_4_) was obtained from Alfa Aesar. Zinc nitrate hexahydrate [Zn(NO_3_)_2_ ∙ 6H_2_O] and hexamethylenetetramine (C_6_H_12_N_4_) were purchased from Aladdin. Sodium dodeyl sulfate (SDS) as the surfactant was obtained from Sinopharm Chemical Reagent Co., Ltd. The precursor materials used for depositing the ZnO layer were diethyl zinc [(C_2_H_5_)_2_Zn, DEZ, Sigma Aldrich, 99.999%] and deionized (DI) water. All chemicals were used as received without any further purification. Other chemical reagents employed in our experiments were analytical grade, and the gases were ultrahigh pure (99.999%). All aqueous solutions were prepared with DI water acquired from a Millipore Q purification system (resistivity >18 MΩ⋅cm).

### Synthesis of highly ordered SnO_2_ nanobowls

The highly ordered SnO_2_ nanobowls were synthesized via a modified hard template method reported before^18^. The suspension of monodispersed PS spheres 800 nm (2.5 wt% in DI water) in diameter was synthesized according to our previous work^50^. Then, the PS suspension was diluted in ethanol of the same volume and subjected to ultrasonic treatment for absolute uniformity. The precursor solution was 0.1 M aqueous SnCl_4_ (100 mL). The ethanol-diluted PS suspension was slowly injected into the precursor solution, and the monolayer PS spheres could float on the surface of the precursor solution and began to self-assemble at the air/solution interface. Specifically, the control of the injection rate was conducted by adjusting the inclination angle of the well-cleaned and oxygen plasma pretreated glass slide, which was applied as a drainage plate. Subsequently, the floating PS monolayer was transferred to the MEMS substrate by a simple picking-up process and thoroughly dried at room temperature. Due to the capillary effect, the PS monolayer on the substrate still retained the precursor solution in the spaces between PS spheres and the substrate^15^. After annealing in the muffle furnace at 550 °C for 2 h, the organic PS template was well removed, and highly ordered SnO_2_ nanobowls were formed in situ on the MEMS substrate.

### Synthesis of highly ordered nanobowl SnO_2_@ZnO films

ZnO seed layers of different thicknesses, namely, 50, 100, and 150 cycles, were deposited at 200 °C on as-prepared highly ordered SnO_2_ nanobowls in a BENEQ TFS-200 ALD system. Briefly, DEZ and DI water were used as the zinc (Zn) and oxidant sources, respectively. The highly ordered SnO_2_ nanobowls were alternately exposed to the vapor pulse of the DEZ and DI water precursors in the ALD reactor chamber using high purity argon gas as the carrier gas. Meanwhile, the high purity Ar as the purge gas purged the gaseous byproducts and residual gas out of the chamber between two valid pulses, effectively avoiding unexpected gas reactions. For all samples, the deposition process in each growth cycle briefly includes a 0.2 s pulse of DEZ, a 10 s purge, a 0.2 s pulse of DI water and a 10 s purge. The different desired thicknesses of ZnO seed layers can be achieved by repeating different specific ALD growth cycles.

### Synthesis of hierarchical highly ordered nanobowl SnO_2_@ZnO NWs

ZnO NWs were grown on highly ordered nanobowl SnO_2_@ZnO films with different ZnO seed layers through a traditional hydrothermal process^51^. In particular, an aqueous solution containing 25 mM Zn(NO_3_)_2_ ∙ 6H_2_O and 25 mM C_6_H_12_N_4_ was prepared as the precursors for ZnO NW growth and transferred into 80 mL Teflon-lined stainless steel autoclaves. Then, highly ordered nanobowl SnO_2_@ZnO films on MEMS substrates were submerged in the solution with face down. The hydrothermal reactions were carried out at 80 °C for different reaction times, namely, 1, 3, 5, and 8 h, and then cooled to room temperature. The substrates were then removed from the solution, rinsed thoroughly with DI water and dried with high pure nitrogen.

### Instruments and characterization

A BENEQ TFS-200 ALD system was used to deposit the ZnO seed layers. The thicknesses of the ZnO films deposited on flat silicon substrates were measured by a SOPRA GES-5E spectroscopic ellipsometry (SE) system. The morphologies of all the samples were recorded on a Zeiss SIGAMA HD field-emission SEM and an FEI Tecnai G^2^ F20 S-TWIN field-emission TEM. Wide-angle XRD patterns were collected on a Bruker D8 Advance powder X-ray diffractometer with Ni-filtered Cu-K_*ɑ*1_ radiation (40 kV, 40 mA, 1.5406 Å). XPS measurements were conducted on a PHI 5000 VersaProbe system using an Mg-K_*α*_ X-ray source.

### Gas sensing performance measurements

For the gas sensing measurements, hierarchical highly ordered nanobowl SnO_2_@ZnO NWs were prepared in situ on MEMS heating appliances. Then, the ultrasonic wire bonding technique was applied to connect the fabricated MEMS device with the external circuit. A JF02F gas sensing measurement system was used to characterize the sensing characteristics of the fabricated hierarchical highly ordered nanobowl SnO_2_@ZnO NW sensors for reducing gases, namely, H_2_S, NH_3_, CH_3_COCH_3_, C_7_H_8_, and HCHO. In this work, for *n*-type SMO materials, the gas sensing response (R) in the reducing-gas measurements was defined as R = R_a_/R_g_, where R_a_ represents the resistance of materials in air and R_g_ represents the resistance in the detecting gas. The response and recovery times were determined as the time required from 10 to 90% of the highest response variation when exposed to the target gas and air, respectively. The measurements under different temperatures were conducted to investigate the optimal operating temperature for our sensors. Moreover, to determine the long-term stability and repeatability, the H_2_S sensing performance of the hierarchical highly ordered nanobowl SnO_2_@ZnO NW sensor preserved in an air atmosphere was characterized again after a month.

## Conclusions

In conclusion, hierarchical highly ordered SnO_2_ nanobowl branched ZnO NWs were synthesized through a sequential process combining hard template processing, atomic-layer deposition and hydrothermal processing. The designed hierarchical sensing materials were prepared in situ on MEMS, which is expected to achieve long-term stability and low power consumption. Specifically, the hierarchical nanobowl SnO_2_@ZnO NW sensor displayed a high sensitivity (R_a_/R_g_) of 6.24, a fast response and recovery speed (i.e., 14 s and 39 s, respectively), and an excellent selectivity when detecting 1 ppm H_2_S at 250 °C, whose rate of resistance change (i.e., 5.24) is 2.6 times higher than that of the pristine SnO_2_ nanobowl sensor. The substantially improved sensing performance could be mainly attributed to the synergistic effect of the hierarchical heterostructures, including increased specific surface area, the formation of heterojunctions and homojunctions, as well as the additional reaction between ZnO and H_2_S. Based on our results, such well-designed hierarchical highly ordered nanobowl SnO_2_@ZnO NW gas sensors are good candidates for enhanced sensitive and selective H_2_S detection with long-term stability and repeatability.

## Supplementary information

Supporting Infromation
