# Supplementary material for: Hierarchical highly ordered SnO2 nanobowl branched ZnO nanowires for ultrasensitive and selective hydrogen sulfide gas sensing
Source: Microsyst Nanoeng. 2020 May 4;6:30. doi: 10.1038/s41378-020-0142-6 (PMC8433378; doi:10.1038/s41378-020-0142-6)
Supplement: Supplementary file 1 — Supporting Infromation [file 41378_2020_142_MOESM1_ESM.docx]

**Hierarchical Highly Ordered SnO_2_ Nanobowls Branched ZnO Nanowires for Ultrasensitive and Selective Hydrogen Sulfide Gas Sensing**

Li-Yuan Zhu ^a^, Kai-Ping Yuan ^a^, Jia-He Yang ^a^, Cheng-Zhou Hang ^a^, Hong-Ping Ma ^a^, Xin-Ming Ji ^a^, Anjana Devi ^b^, Hong-Liang Lu ^a,^*, and David Wei Zhang ^a,^*

*^a^ State Key Laboratory of ASIC and System, Shanghai Institute of Intelligent Electronics & Systems, School of Microelectronics, Fudan University, Shanghai 200433, China*

*^b^Inorganic Materials Chemistry, Ruhr-University Bochum, 44780 Bochum, Germany*

*E-mail: [honglianglu@fudan.edu.cn](mailto:honglianglu@fudan.edu.cn); [dwzhang@fudan.edu.cn](mailto:dwzhang@fudan.edu.cn)

**Supporting Information**


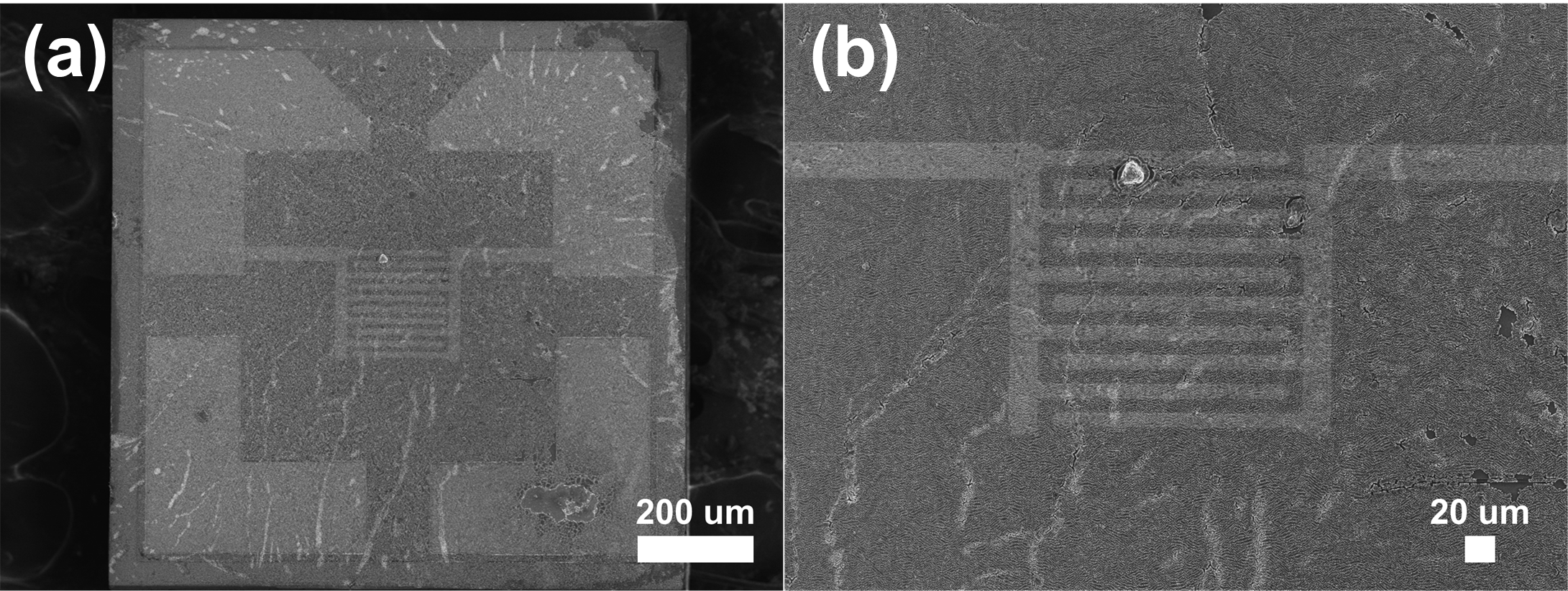


**Figure S1.** SEM characterization of the MEMS substrate with gas sensing materials after 550 ^o^C calcination: (a) low magnification and (b) high magnification.


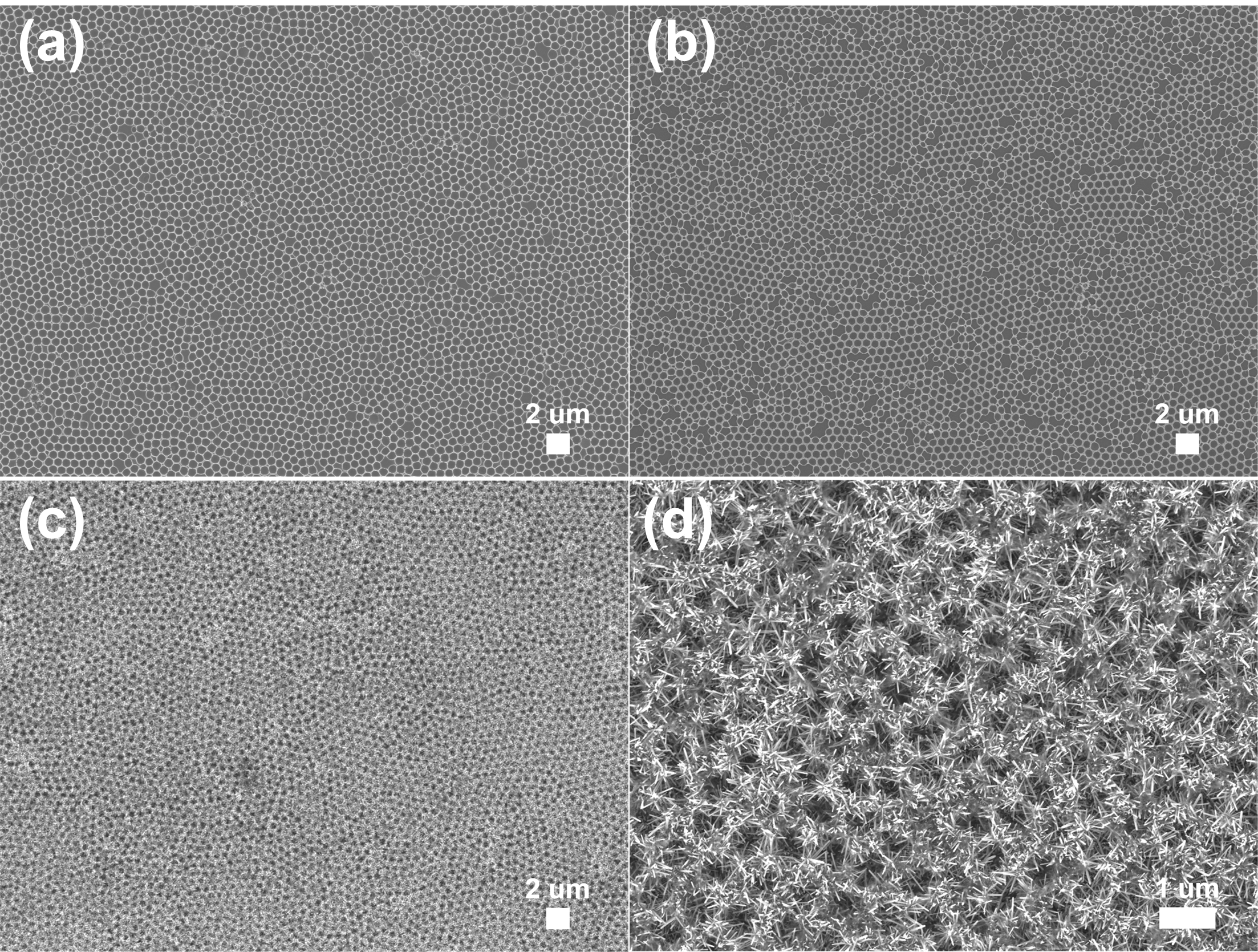


**Figure S2.** SEM characterization with low magnification of (a) pristine highly ordered SnO_2_ nanobowls (*i.e.* S@Z0), (b) highly ordered SnO_2_ nanobowls coated with 20 nm ZnO film (*i.e.* S@Z20), and (c-d) highly ordered SnO_2_ nanobowls branched ZnO NWs (*i.e.* S@Z20-Z5).

As shown in **Figure S2**, the low-magnification SEM images indicate the highly ordered nanobowl structure with a large area. Therefore, the *in situ* preparation method holds the potential for large-scale fabrication.


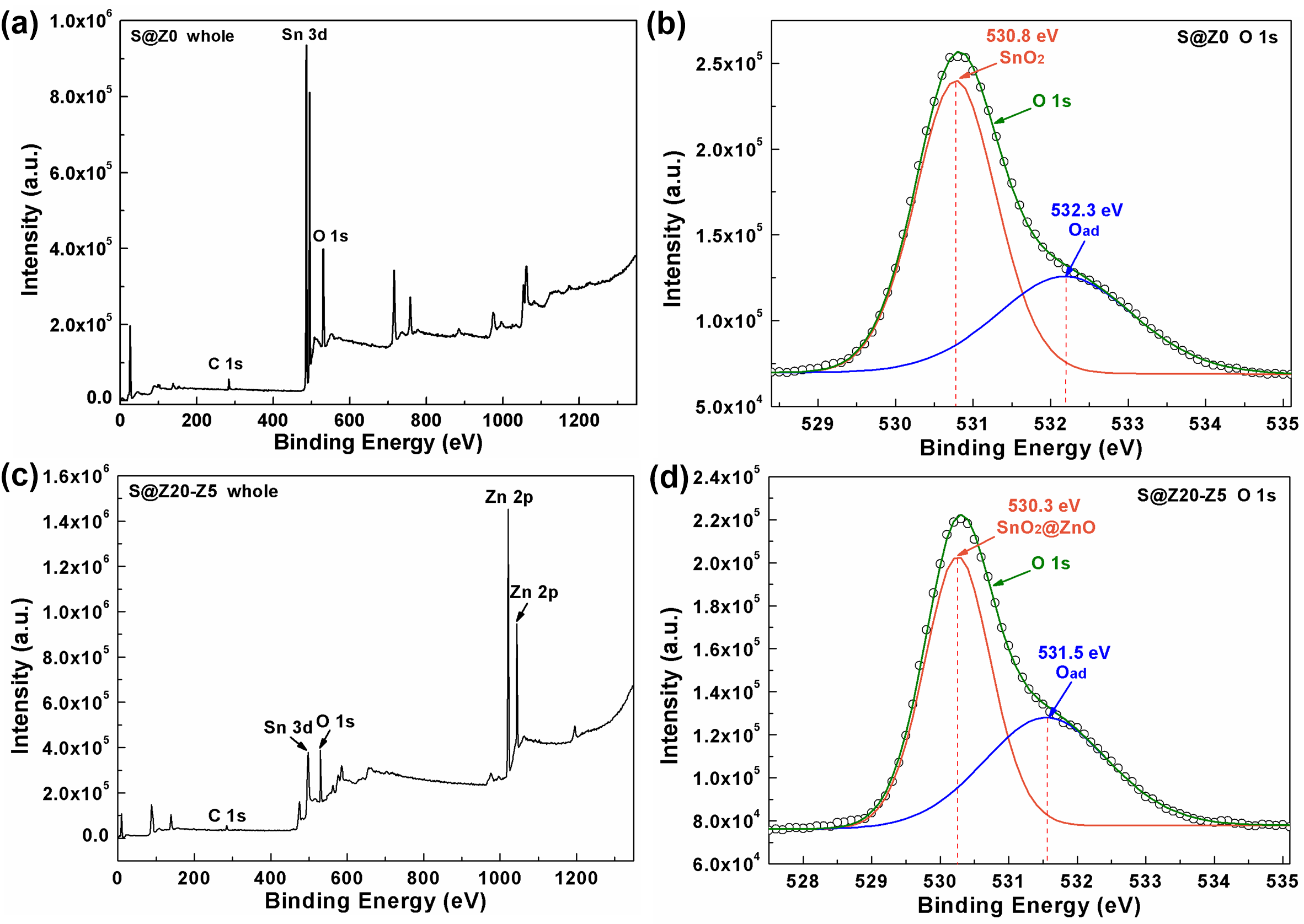


**Figure S3.** (a-b) XPS survey spectra of the highly ordered SnO_2_ nanobowls sample S@Z0, namely (a) full spectrum and (b) O 1s spectrum; (c-d) XPS survey spectra of the highly ordered SnO_2_ nanobowls branched ZnO NWs sample S@Z20-Z5, namely (c) full spectrum and (d) O 1s spectrum.


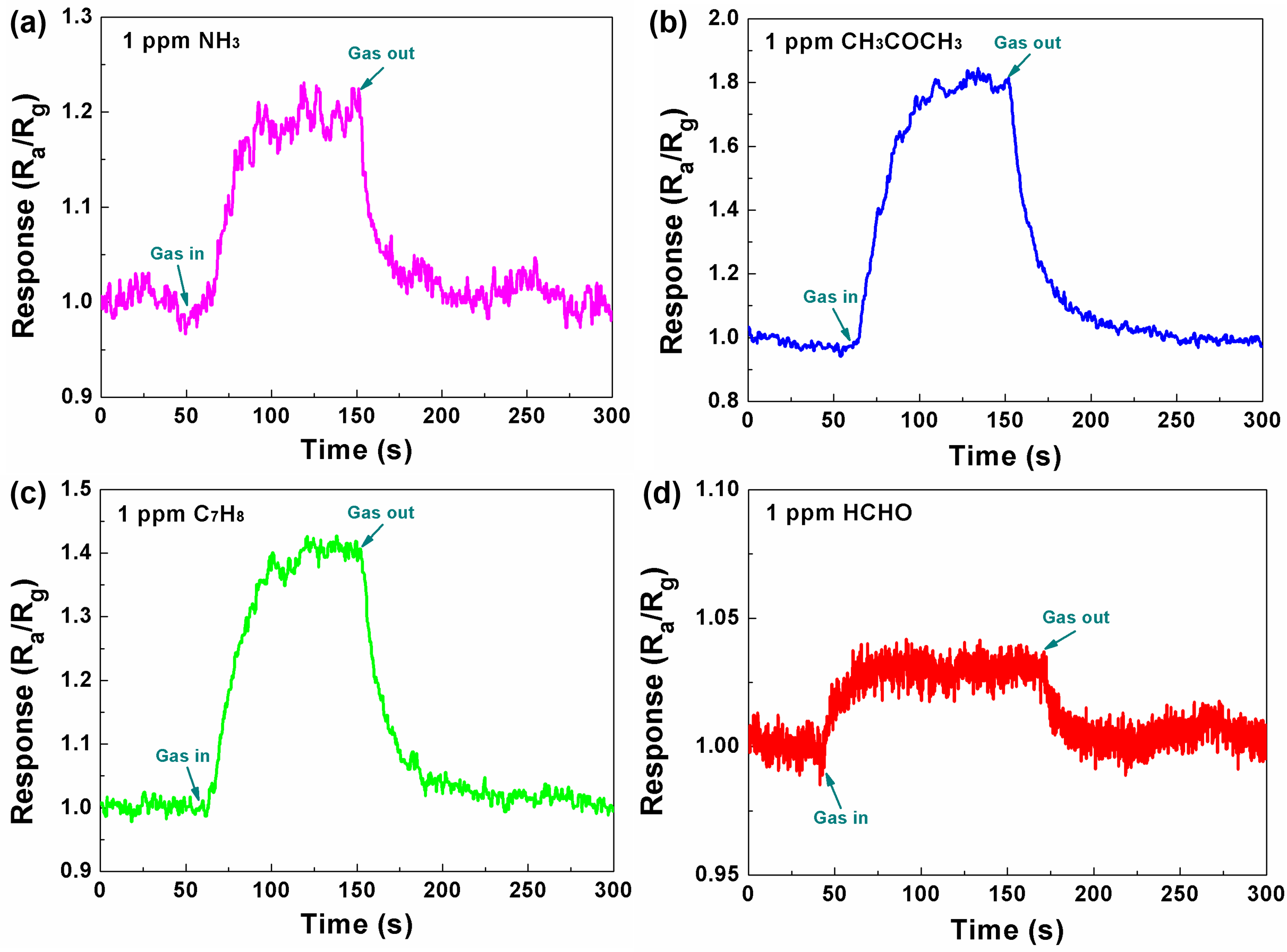


**Figure S4.** Enlarged responses of the sample S@Z20-Z5 facing various reducing gases (1 ppm), namely (a) formaldehyde, (b) acetone, (c) methylbenzene, and (d) ammonia, at the operating temperature of 250 ^o^C.

**Figure S4** displays the responses of the sample S@Z20-Z5 facing 1 ppm NH_3_, CH_3_COCH_3_, C_7_H_8_, and HCHO are 1.20, 1.76, 1.40, and 1.04, respectively. In order to exhibit the apparent variation of every curve, we have chosen different coordinate axis scales in both X and Y directions.


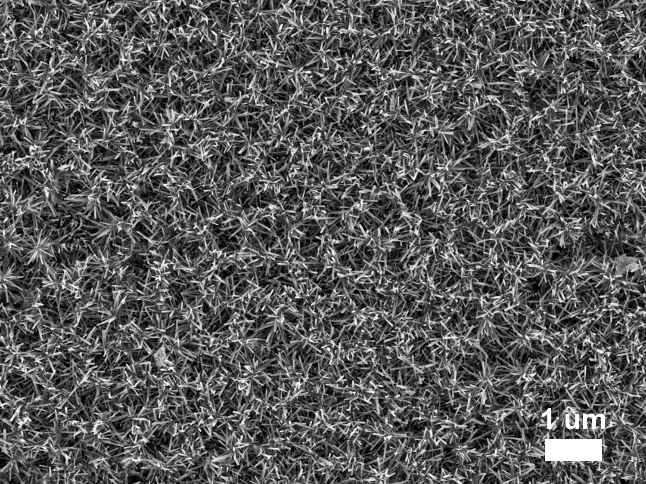


**Figure S5**. The top-view SEM characterization of a randomly selected area of S@Z20-Z5 after long-term gas sensing tests.


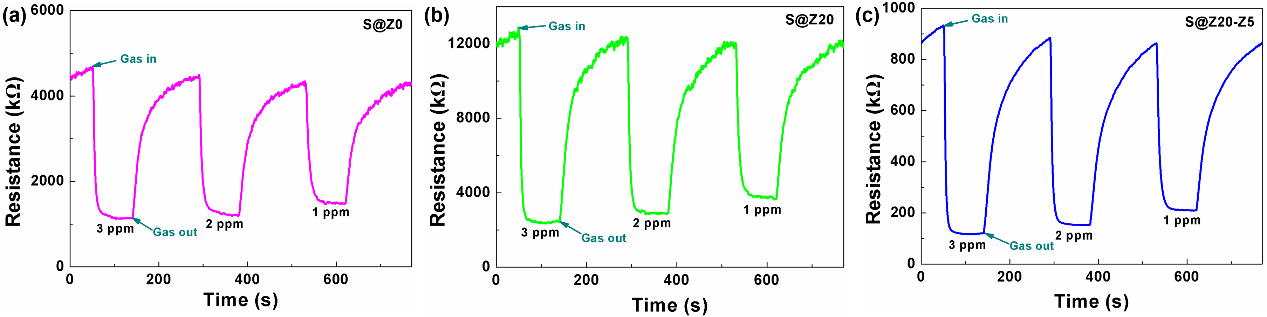


**Figure S6.** Enlarged resistance curves of all the samples, namely (a) S@Z0, (b) S@Z20, and (c) S@Z20-Z5, facing the reducing gas of H_2_S with various concentrations ranging from 3 to 1 ppm under the operating temperature of 250 ^o^C.


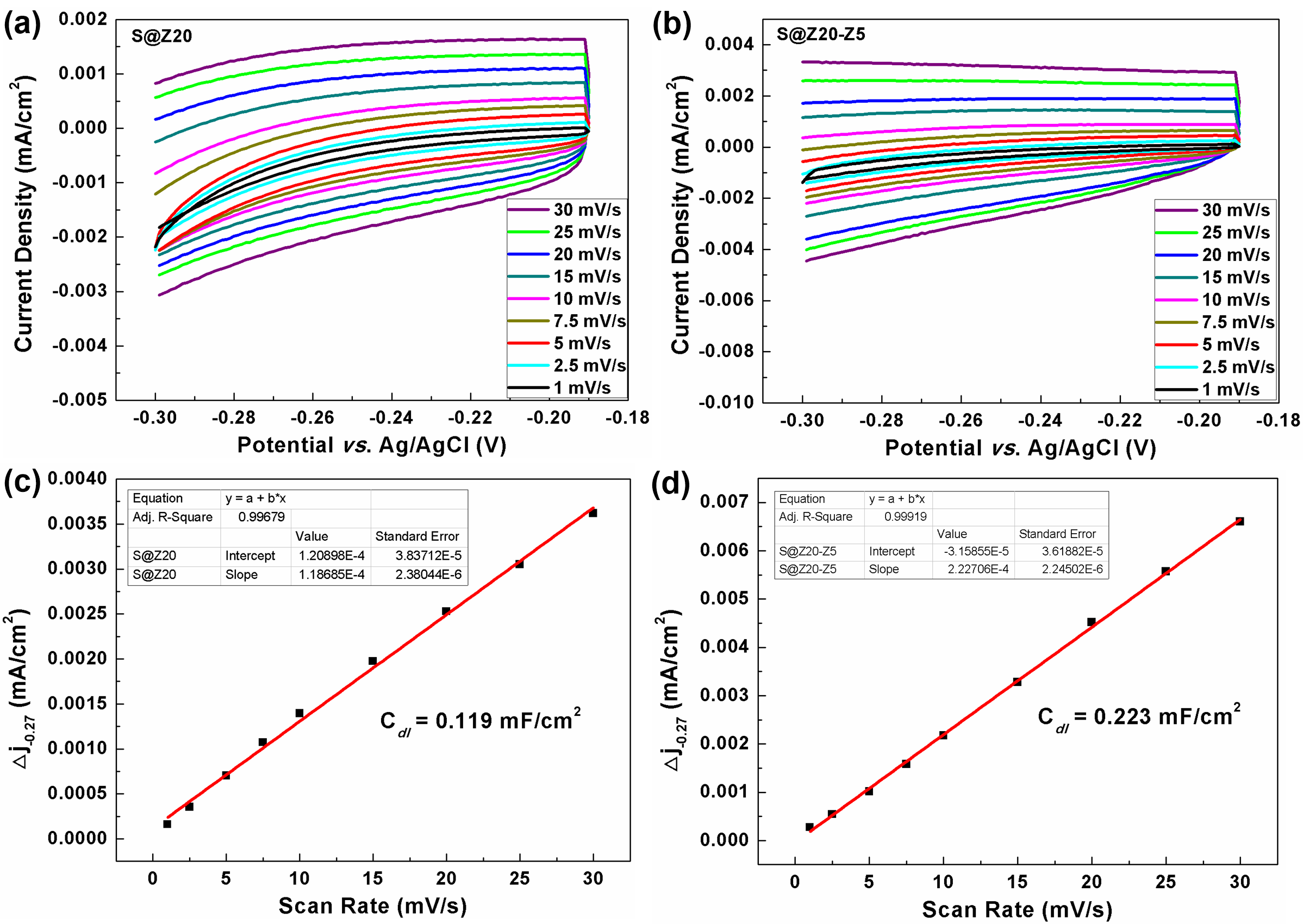


**Figure S7.** (a-b) The cyclic voltammetry curves of (a) S@Z20 and (b) S@Z20-Z5 electrode at various scan rates; (c-d) The capacitive currents at -0.27 V as a function of scan rate for (c) S@Z20 and (d) S@Z20-Z5 electrode (Δ*j = j_a_-j_c_*).
